# Methanotrophic potential of Dutch canal wall biofilms is driven by *Methylomonadaceae*

**DOI:** 10.1093/femsec/fiad110

**Published:** 2023-09-12

**Authors:** Koen A J Pelsma, Daniël A M Verhagen, Joshua F Dean, Mike S M Jetten, Cornelia U Welte

**Affiliations:** Department of Microbiology, Radboud Institute for Biological and Environmental Sciences, Radboud University, Heyendaalseweg 135, 6525 AJ Nijmegen, The Netherlands; Department of Microbiology, Radboud Institute for Biological and Environmental Sciences, Radboud University, Heyendaalseweg 135, 6525 AJ Nijmegen, The Netherlands; School of Geographical Sciences, University of Bristol, Bristol BS8 1SS, United Kingdom; Department of Microbiology, Radboud Institute for Biological and Environmental Sciences, Radboud University, Heyendaalseweg 135, 6525 AJ Nijmegen, The Netherlands; Department of Microbiology, Radboud Institute for Biological and Environmental Sciences, Radboud University, Heyendaalseweg 135, 6525 AJ Nijmegen, The Netherlands

**Keywords:** climate change microbiology, eutrophication, greenhouse gases, *Methyloglobulus*, microbial community, urban waterways

## Abstract

Global urbanization of waterways over the past millennium has influenced microbial communities in these aquatic ecosystems. Increased nutrient inputs have turned most urban waters into net sources of the greenhouse gases carbon dioxide (CO_2_) and methane (CH_4_). Here, canal walls of five Dutch cities were studied for their biofilm CH_4_ oxidation potential, alongside field observations of water chemistry, and CO_2_ and CH_4_ emissions. Three cities showed canal wall biofilms with relatively high biological CH_4_ oxidation potential up to 0.48 mmol g_DW_^−1^ d^−1^, whereas the other two cities showed no oxidation potential. Salinity was identified as the main driver of biofilm bacterial community composition. *Crenothrix* and *Methyloglobulus* methanotrophs were observed in CH_4_-oxidizing biofilms. We show that microbial oxidation in canal biofilms is widespread and is likely driven by the same taxa found across cities with distinctly different canal water chemistry. The oxidation potential of the biofilms was not correlated with the amount of CH_4_ emitted but was related to the presence or absence of methanotrophs in the biofilms. This was controlled by whether there was enough CH_4_ present to sustain a methanotrophic community. These results demonstrate that canal wall biofilms can directly contribute to the mitigation of greenhouse gases from urban canals.

## Teaser

Canal wall biofilms in Dutch urban canals contain methane-oxidizing bacteria that mitigate part of the canal’s methane emissions.

## Introduction

Urban waters are increasingly recognized as important ecosystems that contribute significantly to global greenhouse gas emissions. As even low methane (CH_4_) emissions can have a great impact on climate warming (Myhre et al. 2013), CH_4_ with its high global warming potential has been the focus of several studies of urban ponds (van Bergen et al. 2019, Peacock et al. 2021) and even whole cities (Martinez-Cruz et al. 2017, Herrero Ortega et al. 2019, Wang et al. 2021). While streams, rivers, and lakes are well studied (Bastviken et al. 2011, Stanley et al. 2016, Saunois et al. 2020), urban canals and ditches are poorly represented in recent datasets (Peacock et al. 2021).

Greenhouse gas emissions from aquatic systems are the result of microbial respiration and anaerobic degradation of organic matter (Conrad 2009, Dean et al. 2018). Urban canals are susceptible to many factors that could increase anoxia, and, consequently, higher CH_4_ fluxes, such as slow, laminar water flow and high levels of nutrients (Needelman et al. 2007, Peacock et al. 2021). Lower availability of oxygen causes a larger methanogenic zone and increases CH_4_ emissions. In the Netherlands, many urban waters are considered to have low water quality due to phosphate and ammonia loading, yearly algal blooms, and excessive human activity like boating and recreation (Teurlincx et al. 2019, Armstrong et al. 2022). Therefore, urban canals have the potential to be a substantial source of CH_4_ in the Netherlands (Stanley et al. 2016, Peacock et al. 2021). Due to the extensive use of canals in many Dutch cities, the environmental impact might be considerable but has been poorly constrained.

Methanotrophic bacteria (MOB) and archaea in the sediment or water column can consume CH_4_, acting as a biological filter. In the sediment, anaerobic CH_4_ oxidation (AOM) can occur in freshwaters using a variety of electron acceptors such as NO_3_^−^, NO_2_^−^, iron, and organic matter (Ettwig et al. 2010, Haroon et al. 2013, Ettwig et al. 2016, Valenzuela et al. 2019). For several lakes and streams, this AOM process has been observed to be ecologically relevant (Martinez-Cruz et al. 2018, Shen et al. 2019). The aerobic MOB *Bacillus methanica* was in fact first isolated from an urban canal in Delft by Söhngen (1906), and aerobic methanotrophs are considered to be an important CH_4_ sink at the global scale due to their high CH_4_ conversion rates (Frenzel et al. 1990, Hanson and Hanson 1996, Knief 2015). In lakes and rivers, MOB thrive at the sediment–water interface or in the water column. Several studies have determined the methanotrophic potential of the water column in lakes (*e.g*., Carini et al. 2005, Guérin and Abril 2007, Thottathil et al. 2019, Reis et al. 2020), but for flowing waters, this has been performed only for the rivers Saar and Elbe, Germany (Zaiss et al. 1982, Matoušů et al. 2017), the Yellow River, China (Hao et al. 2020), and the Condamine River, Australia (Burrows et al. 2021).

Recently, a study by Pelsma et al. (2022) reported a novel urban habitat for MOB in the form of the canal wall biofilm. Exposed to both air and water, these biofilms were hypothesized to be an excellent habitat for methanotrophs as they experience little water turbulence and are exposed to high CH_4_ concentrations. The biofilm methanotroph present was *Methyloglobulus morosus*, previously isolated from lake sediment (Deutzmann et al. 2014). However, little is known about methanotrophic biofilms in the built aquatic environment. We hypothesized that MOB are present and metabolically active in a broader range of urban aquatic systems. To test this hypothesis, we sampled canal wall biofilms in five cities in the Netherlands with canals ranging from saline to freshwater. In addition, we investigated the impact of surface material on the microbial community and methanotrophic activity. Lastly, we synthesized field observations and flux data to better identify potential urban hotspots of microbial CH_4_ cycling.

## Materials and methods

### City selection and sample sites

**Figure 1.**
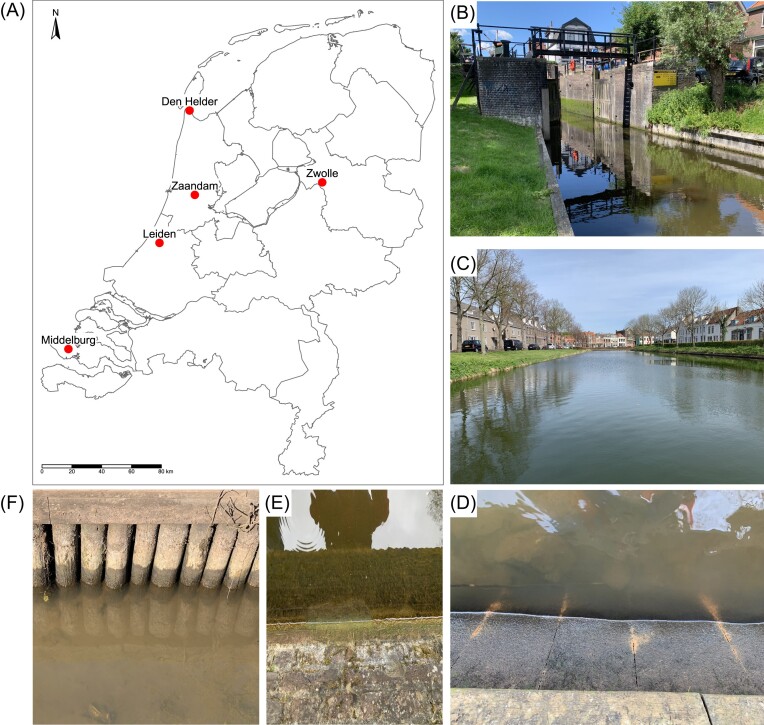
Location of the five sampled cities within the Netherlands (A). Examples of canals in Zaandam (B) and Middelburg (C). Sampled biofilms in Zwolle (F and D) and Leiden (E) showed that there is great diversity in the types of canals and biofilm growth surfaces. An interactive version of a sample site map is available at https://methanecanals.shinyapps.io/data_maps/.

The Netherlands has many cities with urban canals as part of the cityscape. We chose five representative cities along a salinity gradient with different environmental parameters in order to capture several types of biofilms in this cross-sectional study. The city of Middelburg was chosen as it is known to be saline due to its proximity to the Scheldt estuary and North Sea. Den Helder and Zwolle were chosen based on their location in the northern and eastern extremities of the Netherlands. Zaandam, like Amsterdam, is influenced by the saline Noordzeekanaal and has brackish canals. Leiden was chosen to represent a freshwater canal system in the central Netherlands. All cities were sampled between March and July 2022, with a minimum of four sample sites per city (Table 1, Fig. 1; https://methanecanals.shinyapps.io/data_maps/). Sample sites within each city were chosen based on the accessibility to the air–water interface of the canal wall from the street level, whilst also allowing for a dispersed spread of locations. Sampling was undertaken on a single day for each city and samples were processed to a stable state within 24 h after sampling. Maps with sampling sites and photographs of the locations are presented in the Supplementary Materials.

**Table 1.** Sample locations within the cities.

City	Site	Date	Coordinates	Water temperature [°C]	Salinity [µS/cm]	pH	Site description
Zwolle	1	08/03/2022	52°30′57.2′ 6°05′04.9″E	6.3	684	7.36	Shallow broad canal next to a main motorway (A28)
	2	08/03/2022	52°30′51.7′N 6°05′26.4″E	6.8	443	7.93	Main urban canal in the city (Thorbeckegracht)
	3	08/03/2022	52°30′57.6′N 6°05′34.0″E	6.1	437	7.81	A northern canal that used to be part of a defensive moat, now used as a small harbour
	4	08/03/2022	52°30′34.1′N 6°05′39.8″E	7.1	440	7.62	Southern part of the defensive moat, flanked by a busy road to the south and a park
Middelburg	1	11/04/2022	51°29′44.7′N 3°36′33.9″E	11	20 640	8.64	A southern stretch of the Binnengracht, the city’s main canal
	2	11/04/2022	51°30′01.0′N 3°36′30.8″E	11.1	21 170	8.68	A western site of the Binnengracht
	3	11/04/2022	51°30′09.9′N 3°36′38.4″E	12.7	21 460	8.89	The most northern part of the Binnengracht. Here the canal ends
	4	11/04/2022	51°30′03.5′N 3°37′19.7″E	11.5	21 550	8.77	The main harbour of Middelburg, northern part
	5	11/04/2022	51°29′58.9′N 3°37′20.7″E	10.6	22 490	8.59	The main harbour of Middelburg, southern part
	6	11/04/2022	51°30′11.7′N 3°37′34.0″E	12.3	8500	8.54	A suburban ditch that enters the city from the northeast
Leiden	1	25/04/2022	52°09′23.3′N 4°29′10.4″E	12.8	780	8.02	One of the main canals (Rapenburg)
	2	25/04/2022	52°09′45.6′N 4°29′17.4″E	14	770	7.99	A northern central canal (Oude Vest)
	3	25/04/2022	52°09′36.5′N 4°29′45.0″E	12.5	776	7.85	A central canal that used to be the main river supply (Oude Rijn)
	4	25/04/2022	52°09′27.9′N 4°29′31.6″E	14.1	793	7.91	A central canal, next to the city hall (Nieuwe Rijn)
	5	25/04/2022	52°09′21.7′N 4°30′17.8″E	13.2	742	7.76	A canal just east of the main city centre (Rijnkade)
Den Helder	1	31/05/2022	52°57′31.4′N 4°44′52.5″E	18	2960	7.89	A suburban canal, flanked by a grassy shoreline
	2	31/05/2022	52°57′44.7′N 4°45′12.8″E	16.9	3020	8.01	A northern section of the main canal (Kerkgracht)
	3	31/05/2022	52°57′04.7′N 4°46′46.5″E	18	2310	7.94	A southwestern canal now used for houseboats and small quays (Bassingracht)
	4	31/05/2022	52°57′40.1′N 4°46′05.0″E	17.2	2610	7.74	A canal in front of the Dutch maritime museum
Zaandam	1	04/07/2022	52°25′58.4′N 4°50′01.7″E	20.6	9910	7.54	A canal that connects to Zijnkanaal G, in front of the sluice gates (Hanenpadsluis)
	2	04/07/2022	52°25′59.5′N 4°50′04.6″E	20.1	3760	7.51	A canal that connects to Zijnkanaal G, behind the sluice gates (Hanenpadsluis). Here the sluice gates opened and an ebullition event occurred
	3	04/07/2022	52°26′11.3′N 4°49′02.3″E	21.8	1889	7.8	A canal in the west of the city centre in a residential area
	4	04/07/2022	52°26′20.7′N 4°49′10.4″E	21.8	2260	7.57	A central canal in the main shopping area of the city (Gedempte Gracht)

### *In situ* flux measurements and canal water chemical analyses

Diffusive fluxes of CH_4_ and carbon dioxide (CO_2_) were measured using the floating chamber method (Lorke et al. 2015) and a portable greenhouse gas analyser (LI-7810 CH_4_/CO_2_/H_2_O Trace Gas Analyser; LI-COR Inc., USA). Triplicate measurements were done at each site and measurements influenced by gas bubbles were discarded from the analysis. A bubble event was recognized either by eye or when a very sudden CH_4_ spike was detected that went upwards of 5000 ppb. Fluxes were calculated through linear regression of the CH_4_ or CO_2_ concentration over the measurement time (Van Bergen et al. 2019):


(1) \begin{eqnarray*}
{{{F}}}_{{\mathrm{CH}}_{4}} = \frac{{{\mathrm{\Delta ppb}}}}{{{{\Delta \mathit{ t}}}}}\frac{{{{P}}\,{{{M}}}_{{W}}\,{{{V}}}_{{\mathrm{Chamber}}}}}{{{{R}}\,{{T}}\,{\mathrm{Are}}{{\mathrm{a}}}_{{\mathrm{Chamber}}}\,1000}}.
\end{eqnarray*}

*In situ* pH, conductivity, temperature, and dissolved oxygen were measured using a Hach HQ4300 multi-parameter probe (Hach, The Netherlands). Canal water concentrations of NO_3_^−^, NH_4_^+^, and PO_4_^3−^ were measured using colorimetric assays on an AutoAnalyzer3 (Bran+Luebbe, Germany) after filtering 40 ml water using a nylon 0.22 µm syringe filter into a sterile 50 ml centrifuge tube. Samples were stored at −20°C if they were not measured within 1 week. Total organic carbon (TOC) and total nitrogen were measured on a TOC-L CPH/CPN analyser (Shimadzu Benelux, The Netherlands) using unfiltered canal water. Metal concentrations of Cr, Mn, Fe, Co, Ni, Cu, Zn, As, Se, Sr, Mo, Cd, Ba, La, Ce, Nd, Hg, and Pb were measured by inductively coupled plasma mass spectrometry on an Xseries-I (Thermo-Fisher Scientific, Germany). For metals, 10 ml of canal water was acidified with 1% nitric acid (Suprapur, Merck, Germany) to pH ∼2 prior to analysis.

### Biofilm sampling

At each site, three biofilms were scraped from the surroundings and collected in sterile 50 ml centrifuge tubes. Scraping was done using a disposable metal razor that was disinfected using 70% ethanol and air-dried before scraping. For each biofilm subsample, a new razor was used to prevent cross-contamination. Accessibility to the canal wall prevented sampling in some locations, so another static object with visible biofilm growth was sampled instead like a wooden pole or floating pontoons in a harbour. For comparison to the water column, water samples were taken in autoclaved glass bottles by rinsing them with canal water three times and sealing the bottles under water.

### Microcosm CH_4_ oxidation incubations

Oxidation rates of CH_4_ were determined in triplicate per biofilm subsample and were started within 24 h after sampling. Biofilms were distributed equally in autoclaved 120 ml glass serum bottles. Forty millilitre AMS medium (0.5 g l^−1^ NH_4_Cl, 1 g l^−1^ MgSO_4_·7H_2_O, 0.2 gl^−1^ CaCl_2_·2H_2_O, 0.272 g l^−1^ KH_2_PO_4_, and 0.717 g l^−1^ Na_2_HPO_4_·12H_2_O; made in Milli-Q water and autoclaved before adding the phosphate buffer; Whittenbury et al. 1970) supplemented with SL-10 trace elements (German Collection of Microorganisms and Cell Cultures) was used for all biofilm incubations. Additionally, to supply XoxF-type methanol dehydrogenases with lanthanide metals (Keltjens et al. 2014), LaCl_3_ was added to a final concentration of 2 µM. Bottles were capped with grey rubber stoppers and sealed with aluminium crimp caps. While these grey rubber stoppers are less thick than red butyl rubber stoppers, they do not have the potential to inhibit pmoA-based methanotrophy (Niemann et al. 2015). For the canal water incubations, 40 ml of the canal water was used per replicate without any amendments and capped in the same way as the biofilm incubations. Abiotic controls of each biofilm subsample were measured in duplicate by autoclaving the sealed bottles.

A volume of 5 ml of CH_4_ was added to each bottle, after which the bottles were brought up to an overpressure of 0.25 bar by injecting lab air through a 0.22 µm cellulose acetate membrane filters, amounting to a final headspace CH_4_ concentration of 5%. Pressures were checked using a digital pressure meter (GMH 3111, GHM Messtechnik GmbH, Germany). Prior to the first measurement, bottles were left to equilibrate for 3 h at room temperature (21°C). Headspace CH_4_ concentrations were measured daily for at least 2 weeks using 50 µl injections on an HP 5890 Series II (Agilent Technologies, USA) gas chromatograph equipped with a Porapak Q column (80/100 mesh) and a flame ionization detector. Headspace pressures were measured after each measurement day. Using a calibration curve, headspace CH_4_ concentrations were calculated and adjusted using the measured pressures. Oxidation rates were calculated as the slope of a linear regression fitted to data points of the first eight days of incubation. Within this timeframe, biofilm incubations could be considered linear (median *R*^2^ of 0.9). For comparison, biofilms were normalized to gram dry weight (g_DW_) by drying out 10 ml of well-mixed biofilm incubation medium in an 80°C stove.

To determine whether a CH_4_ oxidation rate was due to biological activity or due to other physical effects, all rates were statistically compared to the abiotic control using a Brunner–Munzel test. Only when the median oxidation rate of the biofilms was statistically significant from the abiotic controls (*P* < .05) did we report that a canal wall biofilm can oxidize CH_4_.

### DNA extraction and 16S rRNA gene amplicon sequencing

Biofilm DNA was extracted using the DNeasy PowerSoil DNA extraction kit (Qiagen, The Netherlands) by weighing ∼300 mg in a PowerBead tube. The manufacturer’s instructions were followed except for the homogenization step as this was done using a TissueLyser LT (Qiagen) at 50 Hz for 10 min. DNA was eluted in diethyl pyrocarbonate-treated water (Invitrogen, USA). Eluted DNA was stored at −20°C until sequencing. Due to difficulties during DNA extraction, biofilms from Leiden were not sequenced, and 16S rRNA gene amplicon sequencing was done by Macrogen (Macrogen, Inc., Korea) using the Illumina MiSeq, next-generation sequencing platform. Paired-end libraries were constructed using the Illumina Herculase II Fusion DNA Polymerase Nextera XT Index Kit V2 (Illumina, The Netherlands). Primers used for bacterial amplification were Bac341F (5′-CCTACGGGNGGCWGCAG-3′; Herlemann et al. 2011) and Bac806R (5′-GGACTACHVGGGTWTCTAAT-3′; Caporaso et al. 2012). Raw sequencing reads were deposited in the European Nucleotide Archive at the European Bioinformatics Institute under project number PRJEB62150 (https://www.ebi.ac.uk/ena/browser/view/ PRJEB62150).

### Sequencing data analysis

Raw sequencing reads were filtered and called to amplicon sequence variants (ASVs) using the package *dada2* (Callahan et al. 2016; v1.26) in R4.1.2 (R Core Team 2022). ASVs were assigned taxonomy based on the SILVA SSU 138.1 database (Quast et al. 2012). After trimming, denoizing, dereplication, and chimera removal, each sequencing sample had a minimum 24 103 merged reads with a maximum of 67 592. Biofilm raw reads from the Amsterdam 2019 dataset (Pelsma et al. 2022; PRJEB40426) were re-analysed with the updated packages and added to the analysis. Using the *phyloseq* package (McMurdie and Holmes 2013, v1.42.0), sequence abundance tables were converted to R dataframes. Graphs were constructed using packages available in the package library *tidyverse* (Wickham et al. 2019).

### Statistical analyses

Comparison of CH_4_ oxidation rates to the abiotic control was done using the permuted non-parametric Brunner–Munzel test available in the R package *brunnermunzel* (Neubert and Brunner 2007, Ara 2022). The test was single tailed with a significance of 5% (α = 0.05). Comparison of the biofilm surface type and oxidation potential was done using the Mann–Whitney *U* test through the function *wilcox_test* available in the R package *rstatix* (Kassambara 2023). Correlation analyses on environmental variables were done using Kendall’s τ coefficient. Ordination using non-metric multidimensional scaling (NMDS) was done using the function *metaMDS* from the package *vegan* (Oksanen et al. 2022, v2.6.4). Environmental parameters were tested for significance and fitted to the ordination using *envfit*. All environmental parameters shown were statistically significant after testing with 999 permutations (*p* < .001).

## Results

### Physico-chemical properties of several Dutch urban canals

Five cities with urban canals were sampled to determine the potential of the canal wall biofilm to consume CH_4_. The canals showed large differences in salinity, with Middelburg canals being highly saline, while Leiden and Zwolle had freshwater canals. Zaandam and Den Helder were in between freshwater and brackish canals (Table S1). The canal water temperature increased from Zwolle (6°C) to Zaandam (20°C) because of the time of year during sampling.

Nutrient concentrations differed between cities, with freshwater canals in Zwolle, Den Helder, and Leiden containing increased levels of NO_3_^−^ and NH_4_^+^ up to 90  and 25 µmol l^−1^, respectively. In contrast, the saline canals of Middelburg contained little NO_3_^−^ (0.13–3.53 µmol l^−1^) and NH_4_^+^ (∼3.5 µmol l^−1^). The highest concentrations of PO_4_^3−^ were measured for Den Helder and Zaandam (5.4–29.7 µmol l^−1^), whereas in the other cities, concentrations were below 1 µmol l^−1^. Total organic carbon values ranged from 5.7  to 15.5 mg l^−1^ (Table S1), indicating increased organic carbon loading in the canals, especially in the city of Leiden. The highest iron and cerium concentrations were measured in Zwolle, specifically at site 1 with the lowest pH of 7.36, at 2.98 mg l^−1^. Copper concentrations were highest in Middelburg (saline) with 6.93 µg l^−1^ and Leiden (freshwater) with 9.37 µg l^−1^. Dissolved oxygen was always above 50% air saturation, indicating that anoxia was not occurring in the water columns at the sampling sites.

**Figure 2.**
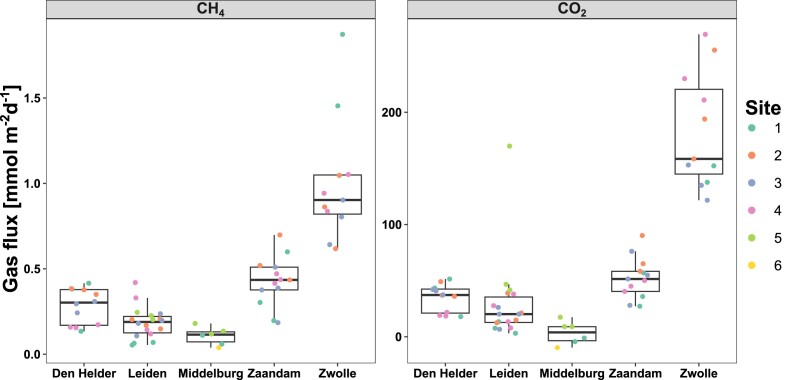
Diffusive flux measurements for CH_4_ (left panel) and CO_2_ (right panel) for the five investigated cities. All individual data points are shown and coloured by sample sites within the city. Note the different *y*-axes for each gas.

Diffusive flux measurements of CH_4_ and CO_2_ showed that every city except Middelburg could be considered a consistent source of CH_4_. For Zaandam and Zwolle, the observed CH_4_ flux was highest at 1.8 and 0.69 mmol m^−2^ d^−1^, respectively (Fig. 2). Both Zwolle’s and Zaandam’s canals were a source of CO_2_ as well at median flux of 158 and 51 mmol m^−2^ d^−1^, respectively. For the saline canals of Middelburg, CH_4_ emissions were only detectable for site 5 (0.14 mmol m^−2^ d^−1^, three replicate measurements), whereas the CO_2_ flux was mostly negative except for site 5 (average of 11.79 mmol m^−2^ d^−1^). Despite high nitrate and TOC, the canals in Leiden did not emit CO_2_ and median CH_4_ emissions were only 0.18 mmol m^−2^ d^−1^. Canals in Den Helder, like Zaandam, were a net source of both CH_4_ and CO_2_ but with some spatial variability within the city.

### CH_4_ oxidation on the canal wall is driven by *Methylomonadaceae*

**Figure 3.**
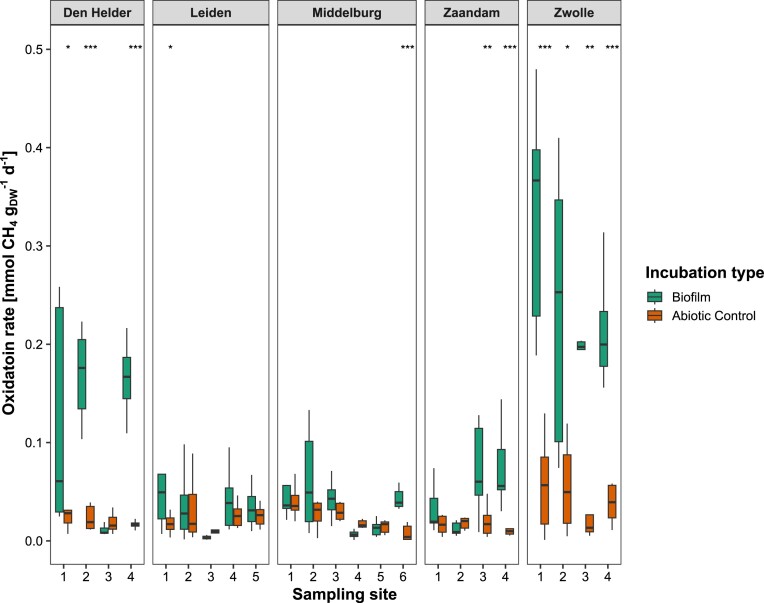
Biofilm methanotrophic rates for each sampling site, ordered by city. Comparison against the abiotic control was done using a Brunner–Munzel test. Per site, nine biofilm incubations were compared against six control incubations. Biofilm incubations with statistically significant differences from the control are marked by an asterisk. *p < .05, **p < .01, and ***p < .001.

Den Helder, Zaandam, and Zwolle hosted canal wall biofilms with clear biological CH_4_ removal (Fig. 3). However, several biofilms, for example, sites 1–5 in Middelburg and sites 2–5 in Leiden, showed CH_4_ oxidation rates of <0.1 mmol g_DW_^−1^ d^−1^, which were not significantly different from the control incubations. The highest observed CH_4_ oxidation rates were 0.48 mmol g_DW_^−1^ d^−1^ in Zwolle (March), 0.31 mmol g_DW_^−1^ d^−1^ in Den Helder (May), and 0.12 mmol g_DW_^−1^ d^−1^ in Zaandam (July). The variability between biofilm subsamples was high as indicated by the large interquartile range (0.01–0.35 mmol g_DW_^−1^ d^−1^ in Zwolle) for the biofilm incubations (Fig. 3). Water column methanotrophy was observed in all cities except Zwolle but with low statistical significance (Fig. S1).

**Figure 4.**
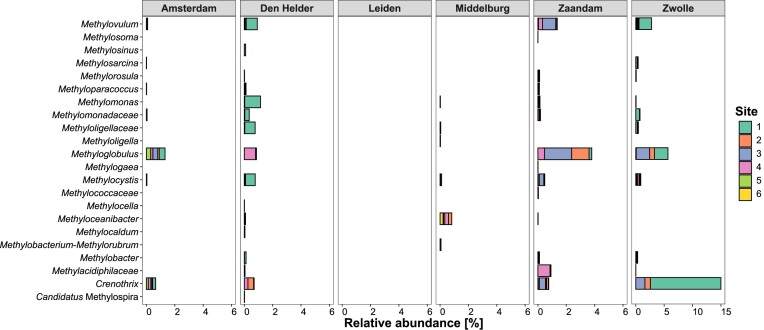
Relative abundances of total bacterial 16S rRNA gene reads of known methanotrophic genera for six sequenced cities. Bars are coloured by sampling site within each city. Leiden biofilms were not sequenced due to DNA extraction difficulties, which is indicated by a blank column. The Amsterdam dataset was obtained from a previous study (Pelsma et al. 2022). Note the different scales for the Zwolle samples.

Biofilm bacterial community profiling showed a dominance of the gammaproteobacterial methanotrophic species *Crenothrix* and *Methyloglobulus* (Fig. 4). Most sampled canal walls were made of either wood or brick. Depending on the city, methanotrophs were more abundant on brick than on wood, but no clear pattern was observed between abundance and canal wall material. In Middelburg, no methanotrophy was observed and the sequencing results indeed showed no reads classified as either *Crenothrix* or *Methyloglobulus*. In Zaandam, these species were observed but only at a relative abundance of 2%, which corresponds quite well to the lower CH_4_ oxidation rates (Fig. 3).

**Figure 5.**
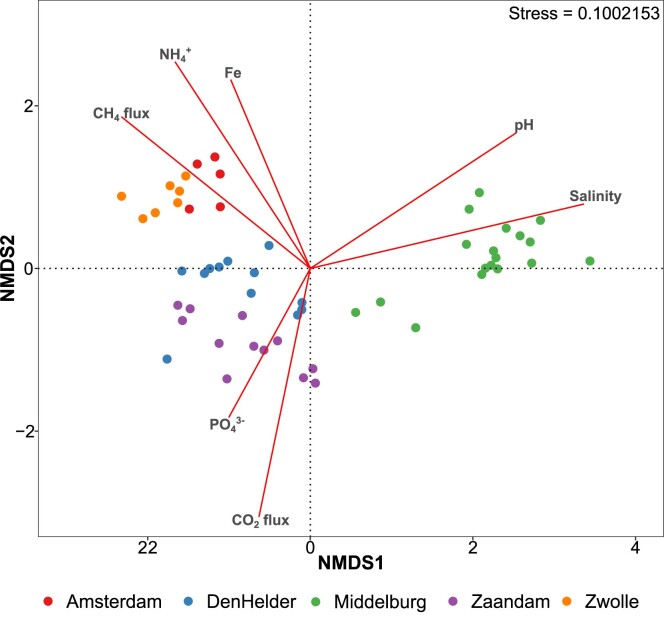
Ordination of the total bacterial community based on 16S rRNA gene amplicon sequencing with Bray–Curtis dissimilarity as distance metric. Environmental variables are scaled based on their effect.

Analysis of the total bacterial community revealed a strong pattern based on salinity and nutrient levels (Fig. 5). High NH_4_^+^ and Fe concentrations in Zwolle clustered it together with Amsterdam (data from Pelsma et al. 2022), while Den Helder and Zaandam also grouped together, separately. Despite differences in NO_3_^−^ concentrations, it did not explain the differences in biofilm community composition as significantly as CH_4_ emissions and PO_4_^3−^. The single brackish site in Middelburg formed a grouping closer to Den Helder and Zaandam, suggesting a stronger influence of salinity than nutrient levels on the biofilm microbial community.

## Discussion

### Regulators of CH_4_ emissions

The potential of urban canals to emit significant amounts of CH_4_ has been well documented in recent years (Stanley et al. 2016, Peacock et al. 2021, Rosentreter et al. 2021, Wang et al. 2021) and the results obtained here support this. Out of the five sampled cities, only the saline canals in Middelburg were not a source of CH_4_. As the canals of Middelburg contained the least amount of dissolved nutrients and were fully oxygenated, they can be considered a marine system. However, during the fieldwork, we observed turbid water with a lot of algal growth in these canals. Due to the time of year we sampled in Middelburg (early April), algal death in late summer could contribute to CH_4_ production and emission in other seasons.

There was some spatial separation of CH_4_ fluxes within the Zwolle city centre, with site 1 having the highest CH_4_ emissions (Fig. 2). This site was characterized by a shallow waterway, so the increase in emissions could be explained by a more turbulent water column that resuspends sediment, likely also causing high dissolved Fe concentrations at this site. However, the other sites were subject to boating, leading to similar resuspension of canal sediment. The NH_4_^+^ and NO_3_^−^ concentrations measured in these canals could contribute to eutrophication of the canal system and subsequent increases in sediment methanogenesis. With our sampling date for Zwolle in early spring, these canals could have higher CH_4_ emissions in late summer not captured by our study. The other freshwater city, Leiden, had substantially lower NH_4_^+^ concentrations and CH_4_ emissions (Fig. 2, Table S1), but higher PO_4_^3−^ concentrations. The sediment floor of these canals was dominated by plants, which could help in aerating the sediment and thereby decreasing methanogenesis in the sediment (Bastviken et al. 2023).

### Biofilm methanotrophy is present in diverse urban canals

The city of Zwolle harboured biofilms with up to 8% methanotrophs, and biological CH_4_ consumption was found at all sites. Compared to the Amsterdam data, we detected similar species but at higher relative abundances and oxidation rates. The absence of methanotrophs from all sampled biofilms in Middelburg in April is consistent with its very low CH_4_ oxidation potential. However, the incubation medium used in this study was not optimized for saline conditions and could have affected the observed methanotrophic rate. Similarly, it has been documented that in the nearby Lake Grevelingen, the relative abundance of methanotrophs and CH_4_ oxidation potential are also lowest around the end of March (Egger et al. 2016, Venetz et al. 2022, Żygadłowska et al. 2023). Only one sampled biofilm from Leiden consumed CH_4_, suggesting it is unlikely that Leiden’s biofilms host methanotrophs.

Biofilm incubations from Zaandam showed that both sites 1 and 2 did not consume CH_4_ and only site 2 harboured *Methylomonadaceae* methanotrophs. These sites are spatially separated from each other by one sluice gate (pictured in Fig. 1b). During the fieldwork, the sluice gate was opened causing a CH_4_ ebullition event. If most CH_4_ is emitted through bubbles, MOB do not have a constant supply of substrate and could be outcompeted by other bacteria in the biofilm. For the other two sites in Zaandam, no ebullition was observed but biofilms did oxidize CH_4_ and harboured methanotrophs, meaning a consistent, if lower, CH_4_ supply is sufficient to sustain a biofilm community with methanotrophic potential. Den Helder was very similar to Zaandam in terms of water chemistry but had much higher oxidation rates. However, for Den Helder site 1, only one subsample had a relative abundance of 9% *Methylomonadaceae*, corresponding to the highest rate. For site 3, no CH_4_ oxidation rate was measured and no MOB could be detected with sequencing (Fig. 4). This site was a small domestic harbour very similar to the other four sites. Therefore, it is evident that within a city canal network, there are local effects of which our study could only capture the heterogeneity to a certain extent. Future research is advised to take spatial heterogeneity into account during study design, as day–night cycles, point sources, and local infrastructure (like sluice gates) can have great effects on both the microbial community and CH_4_ dynamics (Attermeyer et al. 2021, Stanley et al. 2022).

### Conclusions

Our data indicate the presence of biofilm methanotrophy in several distinct urban canal networks across the Netherlands. The two dominant methanotrophs were *Crenothrix* and *Methyloglobulus*, in line with what was previously found in canal biofilms in Amsterdam. The presence of these methanotrophs was associated with consistent diffusive CH_4_ emissions. Their presence could be a way to identify canals with high CH_4_-cycling activity. CH_4_ emissions were observed from most canals and could contribute substantially to total urban CH_4_ emissions. NH_4_^+^ concentration was positively correlated with CH_4_ emissions and CH_4_ oxidation rate, while salinity correlated negatively. We showed that canal wall biofilms may assist in mitigating CH_4_ emissions across a diverse range of Dutch cities. A widespread, year-round monitoring of CH_4_ emissions (diffusive and ebullitive) from urban canals, in conjunction with microbial activity experiments, is required for a better understanding of these ubiquitous and unique Dutch landmarks.

## Supplementary Material

fiad110_Supplemental_Files
