# Supplementary material for: Methanotrophic potential of Dutch canal wall biofilms is driven by Methylomonadaceae
Source: FEMS Microbiol Ecol. 2023 Sep 12;99(10):fiad110. doi: 10.1093/femsec/fiad110 (PMC10561707; doi:10.1093/femsec/fiad110)
Supplement: fiad110_Supplemental_Files [file fiad110_supplemental_files.zip › Supp_data SupplementaryMaterial.docx]

**Supplementary Figures for “Methanotrophic potential of Dutch canal wall biofilms is driven by *Methylomonadaceae*”**

**
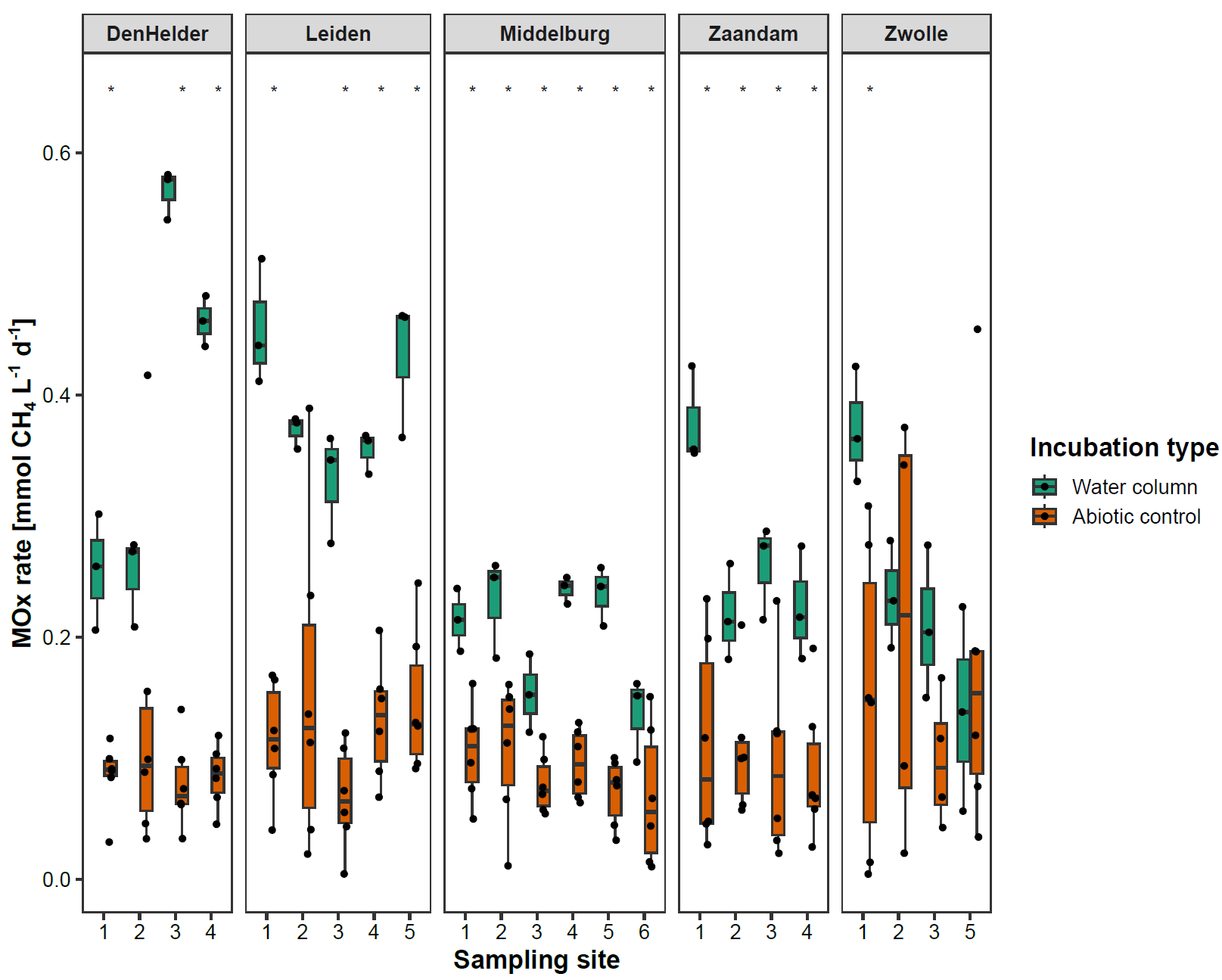
**

**Figure S1.** Water methanotrophic rates for each sampling site, ordered by city. Comparison against the abiotic control was done using a Brunner-Munzel test. Incubations with statistically significant differences from the control are marked by an asterisk. *, *p <* 0*.*05.

**
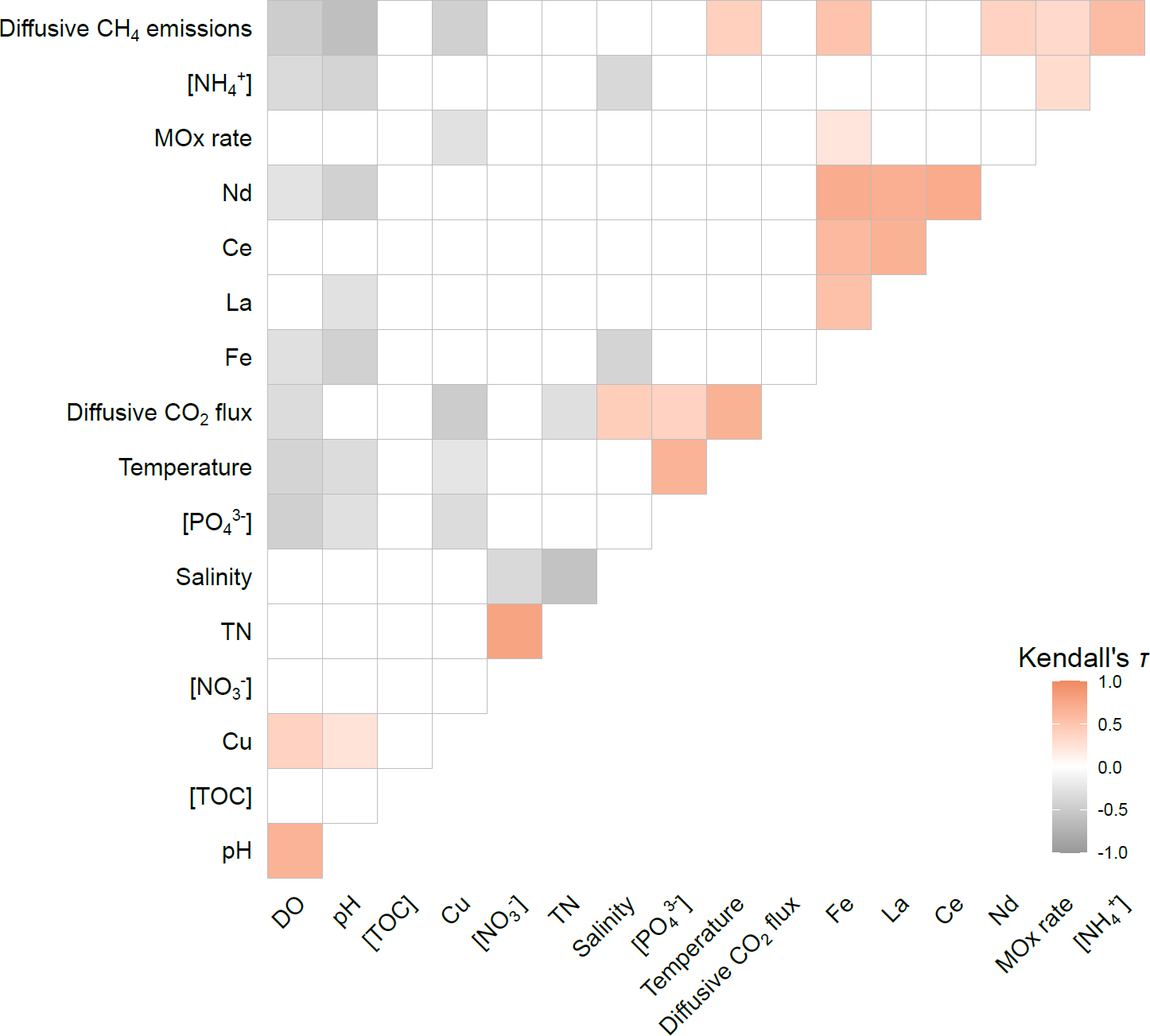
**

**Figure S2.** Correlation matrix of environmental and microbiological parameters measured in this study using Kendall’s *τ* coefficient.
